# Brivaracetam‐associated manic episode in a patient with bipolar disorder: A case report

**DOI:** 10.1002/pcn5.70335

**Published:** 2026-04-27

**Authors:** Mihoko Kawai, Jun Miyata, Kousuke Kanemoto

**Affiliations:** ^1^ Department of Psychiatry Aichi Medical University Nagakute Aichi Japan; ^2^ Epilepsy, Psychiatry Suzukake Clinic Nagoya Aichi Japan

**Keywords:** bipolar disorder, brivaracetam, epilepsy, manic episode, psychiatric adverse events

## Abstract

**Background:**

Levetiracetam and brivaracetam (BRV) are antiseizure medications that exert effects by binding to synaptic vesicle protein 2A (SV2A). BRV exhibits higher affinity and selectivity for SV2A and is generally considered to have a favorable psychiatric tolerability profile. However, reports on serious psychiatric adverse events remain scarce.

**Case Presentation:**

We herein report a 60‐year‐old man with a history of bipolar disorder who had remained psychiatrically stable for many years under aripiprazole treatment. He was diagnosed with focal epilepsy based on stereotyped focal seizures with impaired awareness and right temporal epileptiform discharges observed on electroencephalography. BRV was initiated at a dose of 50 mg/day and later increased to 100 mg/day. Several weeks after initiation, he developed elevated mood, marked irritability, emotional dysregulation, and delusion‐like ideation, progressing to a manic episode with psychotic features requiring inpatient psychiatric care. BRV was discontinued and replaced with lacosamide, coupled with intensification of a mood‐stabilizing treatment, which resulted in the gradual resolution of manic symptoms. No epileptic seizures occurred during BRV treatment or after the medication switch.

**Conclusion:**

This case suggests that, despite its generally favorable psychiatric tolerability, BRV may be associated with the emergence of symptoms in patients with bipolar disorder. These findings highlight the importance of careful psychiatric risk assessment and close monitoring when initiating BRV in individuals with underlying mood vulnerability.

## Graphical abstract

A patient with Bipolar I disorder developed manic symptoms following the initiation of brivaracetam for focal epilepsy. Although brivaracetam is generally considered to have favorable psychiatric tolerability, this case highlights a potential risk of mood destabilization in patients with underlying bipolar disorder.

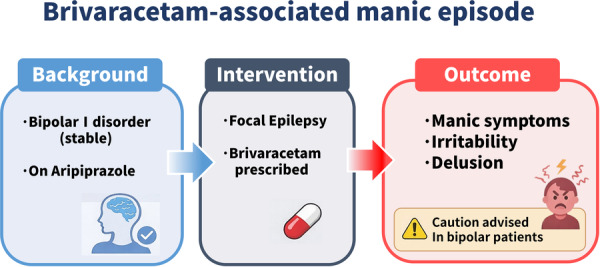

## BACKGROUND

Levetiracetam (LEV) and brivaracetam (BRV) are antiseizure medications widely utilized for focal epilepsy. Both exert primary pharmacological effects by binding to synaptic vesicle protein 2A (SV2A).^1^ SV2A targeting is a mechanism distinct from that of traditional ion channel–blocking antiseizure medications and has been considered to exhibit high antiseizure efficacy with relatively favorable tolerability profiles.^2^

However, LEV is well known to cause behavioral and psychiatric adverse events, including irritability, anger, aggression, depressive symptoms, and psychotic manifestations, which often pose clinical challenges.^3^, ^4^ Essentially, these psychiatric symptoms do not always present as purely depressive states; rather, mood elevation and irritability may predominate, resulting in clinical presentations that resemble mixed affective states from a psychiatric perspective.^5^ Consistent with this observation, sporadic case reports have described the onset of hypomanic or manic episodes after LEV administration.^6^, ^7^

BRV shares the same molecular target as LEV but exhibits higher affinity and selectivity for SV2A. It has generally been reported to cause fewer behavioral and psychiatric adverse effects compared with LEV,^8^, ^9^ and switching from LEV to BRV has been shown to be beneficial in patients who developed such symptoms during LEV treatment.^10^ However, behavioral and psychiatric adverse events can still occur with BRV. Although irritability and behavioral changes have been sufficiently investigated, progression to overt manic or psychotic states has seldom been systematically described, and the clinical features and risk factors underlying these severe manifestations remain poorly understood.

This case report describes a patient with bipolar disorder who had remained psychiatrically stable for many years but developed a distinct manic episode with psychotic features following the initiation and dose escalation of BRV for focal epilepsy. Owing to the scarcity of reports on manic episodes temporally associated with BRV, this case may provide hypothesis‐generating insights into the potential psychiatric effects of SV2A‐targeting antiseizure medications.

## CASE PRESENTATION

The patient was a 60‐year‐old man who had been diagnosed with Bipolar I disorder in his 40s and had a history of psychiatric hospitalization due to a manic episode characterized by elevated mood, grandiose delusions, and pressured speech. After discharge, he continued outpatient treatment with adjustments to his medication. According to the referral documents, his mood condition had remained generally stable for more than 20 years before BRV initiation, with stable adherence to aripiprazole 6 mg/day. Furthermore, he had no history of alcohol or illicit drug use and had not experienced recent mood episodes.

Approximately 1 year before his first visit to our department, his family began noticing episodes of impaired recall of recent conversations and became concerned regarding possible cognitive decline. However, these symptoms did not progress. Approximately 3 months before presentation, the family initially observed stereotyped episodes characterized by sudden staring, followed by oral automatisms and subsequent postictal confusion at a frequency of 2–3 times per month. Approximately 1 month before presentation, a similar episode occurred while he was driving, which resulted in a single‐vehicle accident. Following this event, he visited our department for further evaluation.

**Figure 1:**
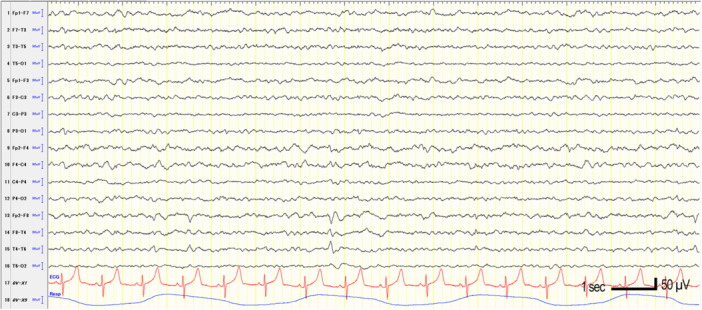
Interictal electroencephalography findings at the initial presentation. Interictal scalp electroencephalography showed recurrent epileptiform discharges predominantly in the right temporal region. These findings are consistent with the diagnosis of focal epilepsy based on the patient's stereotyped focal impaired awareness seizures.

**Figure 2:**
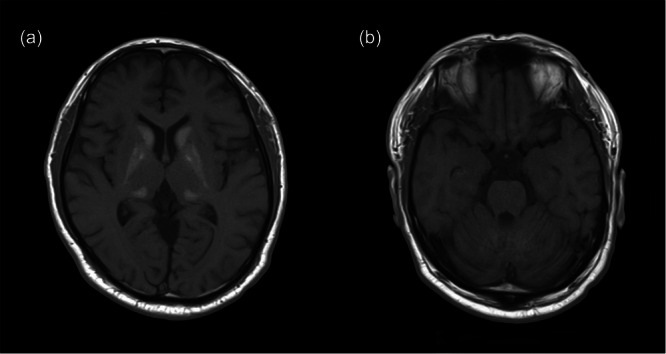
Brain magnetic resonance imaging findings. Fluid‐attenuated inversion recovery (FLAIR) images show (a) symmetrical hypointense lesions corresponding to calcifications in the bilateral basal ganglia, thalami, and cerebellar dentate nuclei, consistent with chronic intracranial calcifications related to idiopathic hypoparathyroidism and (b) no hippocampal atrophy or abnormal signal intensity.

Interictal electroencephalography (EEG) showed recurrent epileptiform discharges in the right temporal region (Figure 1). Brain magnetic resonance imaging revealed no hippocampal atrophy, abnormal signal intensity, or intracranial space‐occupying lesions (Figure 2). Incidentally, symmetrical calcifications were found in the bilateral basal ganglia, thalami, and cerebellar dentate nuclei. Blood tests indicated hypocalcemia, and further evaluation led to a diagnosis of idiopathic hypoparathyroidism. Treatment was initiated by the endocrinology department.

Based on the seizure semiology and EEG findings, a diagnosis of focal epilepsy was made, and BRV was initiated at 50 mg/day. Seizure episodes recurred following the treatment initiation, and with the family's heightened concern after the vehicular accident, the BRV dose was cautiously increased to 100 mg/day approximately 4 weeks later. Retrospectively, family members reported that subtle mood instability may have already been present around 3 weeks following BRV initiation. However, at the scheduled outpatient visit prior to dose escalation, neither the patient nor his family reported overt mood symptoms, and no clear affective disturbance occurred during clinical assessment.

Approximately 1 week after the dose escalation, marked mood elevation, increased irritability, and emotional instability abruptly developed. The patient became excessively reactive to comments from family members and friends and made distressing statements, such as “the world is falling apart.” During an unscheduled outpatient visit, he showed pronounced agitation, intermittently shouting at medical staff while suddenly bursting into tears, suggesting severe difficulty in emotional regulation. He was judged unable to continue living at home; consequently, he was admitted for inpatient psychiatric care, with the consent of his sister.

After admission, aggressive behavior toward other patients and staff persisted, rendering management in a general ward difficult and necessitating care in a seclusion room. Due to the suspicion of BRV‐related psychiatric adverse effects, BRV was discontinued and replaced with lacosamide. However, manic symptoms persisted, and discharge from the seclusion room was initially impossible. Aripiprazole was gradually increased to a maximum dose of 30 mg/day, allowing the discontinuation of seclusion; however, mood elevation persisted. Valproate at 400 mg/day was therefore added, after which the mood symptoms gradually improved.

After treatment adjustment, his general condition stabilized. Epileptic seizures did not recur after the switch to lacosamide, and follow‐up EEG findings revealed an improvement. The Young Mania Rating Scale score improved from 42/60 at admission to 8/60 following discharge from the seclusion room and further to 1/60 at discharge, by which time his manic symptoms had almost completely resolved. Although the symmetrical intracranial calcifications associated with idiopathic hypoparathyroidism may have contributed to seizure susceptibility, a definitive causal relationship could not be established. Therefore, the antiseizure and endocrine treatments were continued in parallel.

## DISCUSSION

Although BRV is generally regarded as a well‐tolerated antiseizure medication with a favorable psychiatric profile, the present case highlights an important clinical exception. In a patient with long‐standing bipolar disorder who had been psychiatrically stable for many years, BRV treatment was temporally linked to the onset of a clear manic episode with psychotic features. This finding challenges the implicit assumption that BRV is universally safe from a psychiatric perspective and suggests that, in susceptible individuals, SV2A‐targeting antiseizure medications may be associated with mood destabilization beyond nonspecific behavioral changes, with potential implications for clinical decision‐making. The prolonged psychiatric stability for over two decades prior to the initiation of BRV strengthens the temporal association, although a definitive causal relationship cannot be established.

To our knowledge, comprehensive clinical descriptions of manic episodes temporally associated with BRV—including seizure semiology, psychiatric manifestations, and treatment course—are exceedingly limited. Accordingly, the current report represents one of the few cases describing the clinical features of manic episodes temporally associated with BRV.

LEV is well known to exert a range of psychiatric adverse effects, including irritability, anger, aggression, depressive symptoms, and psychotic manifestations.^3^, ^4^ From a psychiatric perspective, these symptoms frequently present not as purely depressive states but as irritability, emotional lability, and mood elevation, thereby resulting in a mixed affective–like presentation.^11^ Previous studies have reported that LEV is associated with increased aggression and irritability compared with other antiseizure medications and that these behavioral changes have been objectively quantified using standardized assessment instruments.^12^, ^13^ Although BRV is generally considered to be associated with fewer psychiatric adverse effects than LEV,^8^, ^9^ observational studies and real‐world data indicate that BRV‐related behavioral and psychiatric symptoms, while infrequent, occur.^10^, ^14^, ^15^ The present case suggests that, in patients with underlying mood regulation vulnerability, such as bipolar disorder, these adverse effects may manifest as a distinct manic episode rather than nonspecific behavioral changes.

BRV has been reported to exert fewer off‐target effects on AMPA receptors and voltage‐gated calcium channels than LEV, which may contribute to its favorable psychiatric tolerability profile.^8^ However, the present case indicates that such a mechanism alone may be insufficient to explain SV2A ligand–associated psychiatric adverse effects. Bipolar disorder is characterized by enduring vulnerability in affective regulation networks, even during euthymic states.^16^ In this context, the pharmacological modulation of synaptic transmission by SV2A‐targeting agents such as BRV may interact with preexisting affective regulatory vulnerability, potentially lowering the threshold for mood destabilization.

The psychiatric adverse effects of LEV are known to occur independently of dose,^3^ and the dose–response relationship for BRV‐related psychiatric adverse events remains unclear.^10^, ^17^ In the present case, although manic symptoms became clinically evident during treatment, retrospective data suggested that subtle mood instability may have already been present before the dose escalation; therefore, a clear dose‐dependent relationship could not be established.

Postictal psychosis was considered to be a differential diagnosis; however, no distinct seizure cluster preceded the onset of psychiatric symptoms, and a characteristic lucid interval did not occur. Moreover, psychiatric symptoms emerged independently of seizure activity. In addition, hypocalcemia and symmetrical intracranial calcifications associated with idiopathic hypoparathyroidism were incidentally identified.^18^, ^19^ However, the patient had no prior history of mood destabilization related to metabolic abnormalities, and the seizure semiology was not typical of metabolic seizures, which typically present as generalized convulsive events. While intracranial calcifications may contribute to background vulnerability in neuronal excitability, their causal relationship with focal epilepsy and acute psychiatric destabilization remains uncertain and is largely based on case‐level evidence. The close temporal relationship between symptom onset and BRV initiation and escalation is suggestive of a possible drug‐related effect.

## CONCLUSION

Although BRV is generally considered to be a well‐tolerated antiseizure medication, this case suggests that manic episodes may occur in patients with underlying mood regulation vulnerability. In individuals with a history of bipolar disorder or other mood disorders, careful monitoring of psychiatric symptoms during BRV initiation and dose escalation is warranted. This case underscores the importance of individualized psychiatric risk assessment when selecting SV2A‐targeting antiseizure medications. Further systematic investigation is needed to clarify the risk factors and mechanisms underlying mood‐elevating psychiatric adverse effects associated with LEV and BRV.

## AUTHOR CONTRIBUTIONS

Mihoko Kawai, the corresponding author, confirms that all authors contributed to the conception, design, data acquisition, analysis, and manuscript drafting. All authors reviewed and approved the final version, agreeing to take responsibility for the accuracy and integrity of the work. Although all authors contributed, specific responsibilities were as follows:

As the principal investigator, Mihoko Kawai, had full access to all data and ensured data integrity and analysis accuracy. Mihoko Kawai developed the study concept and design, recruited the patient along with Jun Miyata and Kousuke Kanemoto, created the table, and drafted the manuscript.

## CONFLICT OF INTEREST STATEMENT

The authors declare no conflicts of interest.

## ETHICS APPROVAL STATEMENT

This study was conducted in accordance with the Declaration of Helsinki and was approved by the Ethics Committee of Aichi Medical University (Approval No. 2025‐278).

## PATIENT CONSENT STATEMENT

Written informed consent and a signed release were obtained from the patient.

## CLINICAL TRIAL REGISTRATION

Not applicable.

## Data Availability

The data that support the findings of this study are available from the corresponding author upon reasonable request.

## References

[1] Lynch BA , Lambeng N , Nocka K , Kensel‐Hammes P , Bajjalieh SM , Matagne A , et al. The synaptic vesicle protein SV2A is the binding site for the antiepileptic drug levetiracetam. Proc Natl Acad Sci. 2004;101(26):9861–9866. 10.1073/pnas.0308208101 15210974 PMC470764

[2] Löscher W , Gillard M , Sands ZA , Kaminski RM , Klitgaard H . Synaptic vesicle glycoprotein 2A ligands in the treatment of epilepsy. CNS Drugs. 2016;30(11):1055–1077. 10.1007/s40263-016-0384-x 27752944 PMC5078162

[3] Mula M , Trimble MR , Yuen A , Liu RSN , Sander JWAS . Psychiatric adverse events during levetiracetam therapy. Neurology. 2003;61(5):704–706. 10.1212/01.wnl.0000078031.32904.0d 12963770

[4] Tao K , Chen H , Chen Y , Gu Y , Wang X . Levetiracetam induces severe psychiatric symptoms in people with epilepsy. Seizure. 2024;116:147–150. 10.1016/j.seizure.2022.12.002 36535885

[5] Mula M , Sander JW . Negative effects of antiepileptic drugs on mood in patients with epilepsy. Drug Saf. 2007;30(7):555–567. 17604407 10.2165/00002018-200730070-00001

[6] Mula M , Monaco F . Antiepileptic drug‐induced mania in patients with epilepsy: what do we know? Epilepsy Behav. 2006;9(2):265–267. 10.1016/j.yebeh.2006.06.016 16887395

[7] Youroukos S , Lazopoulou D , Michelakou D , Karagianni J . Acute psychosis associated with levetiracetam. Epileptic Disord. 2003;5(2):117–119. 12875956

[8] Klein P , Bourikas D . Narrative review of brivaracetam: preclinical profile and clinical benefits in the treatment of patients with epilepsy. Adv Ther. 2024;41(7):2682–2699. 10.1007/s12325-024-02876-z 38811492 PMC11213745

[9] Yates SL , Fakhoury T , Liang W , Eckhardt K , Borghs S , D'Souza J . An open‐label, prospective, exploratory study of patients with epilepsy switching from levetiracetam to brivaracetam. Epilepsy Behav. 2015;52(Pt A):165–168. 10.1016/j.yebeh.2015.09.005 26432008

[10] Steinhoff BJ , Klein P , Klitgaard H , Laloyaux C , Moseley BD , Ricchetti‐Masterson K , et al. Behavioral adverse events with brivaracetam, levetiracetam, perampanel, and topiramate: a systematic review. Epilepsy Behav. 2021;118:107939. 10.1016/j.yebeh.2021.107939 33839453

[11] Mula M , Monaco F . Antiepileptic drugs and psychopathology of epilepsy: an update. Epileptic Disord. 2009;11(1):1–9. 10.1684/epd.2009.0238 19258231

[12] Kawai M , Goji H , Kanemoto K . Aggression as psychiatric side effect of newer AEDs in patients with epilepsy: cross‐sectional study based on Buss‐Perry Aggression Questionnaire. Epilepsy Behav. 2021;115:107546. 10.1016/j.yebeh.2020.107546 33444989

[13] Kawai M , Goji H , Kanemoto K . Differences in aggression as psychiatric side effect of levetiracetam and perampanel in patients with epilepsy. Epilepsy Behav. 2022;126:108493. 10.1016/j.yebeh.2021.108493 34933187

[14] Villanueva V , Laloyaux C , D'Souza W , Faught E , Klein P , Reuber M , et al. Effectiveness and tolerability of 12‐month brivaracetam in the real world: EXPERIENCE, an international pooled analysis of individual patient records. CNS Drugs. 2023;37(9):819–835. 10.1007/s40263-023-01033-4 37684497 PMC10501958

[15] Szaflarski JP , Besson H , D'Souza W , Faught E , Klein P , Reuber M , et al. Effectiveness and tolerability of brivaracetam in patients with epilepsy stratified by comorbidities and etiology in the real world: 12‐month subgroup data from the international EXPERIENCE pooled analysis. J Neurol. 2024;271(6):3169–3185. 10.1007/s00415-024-12253-z 38436680 PMC11136785

[16] Phillips ML , Swartz HA . A critical appraisal of neuroimaging studies of bipolar disorder: toward a new conceptualization of underlying neural circuitry and a road map for future research. Am J Psychiatry. 2014;171(8):829–843. 10.1176/appi.ajp.2014.13081008 24626773 PMC4119497

[17] Zhu L , Chen D , Chen T , Xu D , Chen S , Liu L , et al. The adverse event profile of brivaracetam: a meta‐analysis of randomized controlled trials. Seizure. 2017;45:7–16. 10.1016/j.seizure.2016.11.008 27898363

[18] Bilezikian JP , Khan A , Potts JT , Brandi ML , Clarke BL , Shoback D , et al. Hypoparathyroidism in the adult: epidemiology, diagnosis, pathophysiology, target‐organ involvement, treatment, and challenges for future research. J Bone Miner Res. 2011;26(10):2317–2337. 10.1002/jbmr.483 21812031 PMC3405491

[19] Saleem S , Aslam HM , Anwar M , Anwar S , Saleem M , Saleem A , et al. Fahr's syndrome: literature review of current evidence. Orphanet J Rare Dis. 2013;8(1):156. 10.1186/1750-1172-8-156 24098952 PMC3853434

